# Evaluation of Antibacterial Activity of Zinc-Doped Hydroxyapatite Colloids and Dispersion Stability Using Ultrasounds

**DOI:** 10.3390/nano9040515

**Published:** 2019-04-02

**Authors:** Daniela Predoi, Simona Liliana Iconaru, Mihai Valentin Predoi, Mikael Motelica-Heino, Regis Guegan, Nicolas Buton

**Affiliations:** 1 National Institute of Materials Physics, 405A Atomistilor Street, P.O. Box MG7, 077125 Magurele, Romania; simonaiconaru@gmail.com; 2 University Politehnica of Bucharest, BN 002, 313 Splaiul Independentei, Sector 6, 060042 Bucharest, Romania; predoi@gmail.com; 3 Institut des Sciences de la Terre D’Orleans (ISTO), UMR, 327, Centre National de la Recherche Scientifique CNRS Université d’Orléans, 1A rue de la Férollerie, CEDEX 2, 45071 Orléans, France; mikael.motelica@univ-orleans.fr; 4 Faculty of Science and Engineering, Global Center for Science and Engineering, Waseda University, 3-4-1 Okubo, Shinjuku-ku, Tokyo 169-8555, Japan; regis.guegan@aoni.waseda.jp; 5 HORIBA Jobin Yvon S.A.S., 6-18 Rue du Canal, CEDEX 91165 Longjumeau, France; nicolas.buton@horiba.com

**Keywords:** zinc-doped hydroxyapatite, nanoparticles, ultrasound technique, antibacterial activity

## Abstract

This study proves that the new developed zinc-doped hydroxyapatite (ZnHAp) colloids by an adapted sol-gel method can be widely used in the pharmaceutical, medical, and environmental industries. ZnHAp nanoparticles were stabilized in an aqueous solution, and their colloidal dispersions have been characterized by different techniques. Scanning Electron Microscopy (SEM) was used to get information on the morphology and composition of the investigated samples. Energy-dispersive X-ray spectroscopy (EDX) analysis confirmed the elemental compositions of ZnHAp colloidal dispersions. The homogeneous and uniform distribution of constituent elements (zinc, calcium, phosphorus, oxygen) was highlighted by the obtained elemental mapping results. The X-ray diffraction (XRD) results of the obtained samples showed a single phase corresponding to the hexagonal hydroxyapatite. The characteristic bands of the hydroxyapatite structure were also evidenced by Fourier-transform infrared spectroscopy (FTIR) analysis. For a stability assessment of the colloidal system, ζ-potential for the ZnHAp dispersions was estimated. Dynamic light scattering (DLS) was used to determine particles dispersion and hydrodynamic diameter (D_HYD_). The goal of this study was to provide for the first time information on the stability of ZnHAp particles in solutions evaluated by non–destructive ultrasound-based technique. In this work, the influence of the ZnHAp colloidal solutions stability on the development of bacteria, such as *Escherichia coli* (*E. coli*) and *Staphylococcus aureus* (*S. aureus*), was also established for the first time. The antimicrobial activity of ZnHAp solutions was strongly influenced by both the stability of the solutions and the amount of Zn.

## 1. Introduction

Over the last decades, due to the population aging and the increase in bone-related affections, as well as the multitude of degenerative diseases and recurrence injuries, the need for effective regenerative or replacement tissues has become pronounced worldwide. These facts have driven both the scientific and industrial communities to focus their attention on developing successful and cost-effective materials that could, in the medical field, replace damaged tissue, organs and also improve their functions [1,2,3].

One of the most studied materials with suitable biocompatibility and osteoconductivity properties used in bone reconstruction is hydroxyapatite (HAp) [3,4]. Synthetic hydroxyapatite, Ca_10_(PO_4_)_6_(OH)_2_, is similar to the human hard tissue and due to its outstanding biocompatibility and osteoconductive properties, it has been successfully used in dentistry, reconstruction surgery, repair surgery, dental implantology, pharmacy, cosmetics, food industry, etc. [4,5,6,7,8]. Hydroxyapatite is also known for its use as a coating for different medical devices [9]. Nowadays, one of the major problems in the area of medical implants is represented by the apparition of post-operatory infections caused by bacterial contamination due to the adherence and colonization of bacterial cells on the biomaterial surface [10]. These types of infections are often caused by antibiotic-resistant bacterial strains, and conventional antibiotic therapy is not always efficient, thereby increasing the rate of morbidity and mortality and health care costs [10]. In order to minimalize the risk of implant-related infections, many authors have encouraged the improvement of the biomaterial used as a coating with different antibacterial agents [5]. Recent studies have reported the use of certain ions, including silver (Ag^+^) [11,12], copper (Cu^2+^) [13], and zinc (Zn^2+^) [14,15], to create hydroxyapatite with antibacterial properties [5]. Zinc is one of the essential microelements found in the human body and is also involved in the metabolism of bones. Zinc ions have also been reported to possess antibacterial activity and to be involved in the proliferation of osteoblast cells [16]. Therefore, developing hydroxyapatite doped with zinc ions will allow the obtaining of a material with both biocompatible and osteoconductive properties, having the capability to stimulate the osteointegration of bones and possessing antimicrobial activity.

Nowadays, the rapid progress of the industrialization process and the competitive market has imposed the need of improved products and better productivity, which has led to more severe expectations for process and quality control in the industry of material science. In this context, material characterization has an important role in the development of new materials and essentially comprises the evaluation of a material’s structure, morphological features, associated mechanical properties, and the evaluation of its elastic behavior. The characterization techniques used for materials analysis are the basic tools for quality control, and the quality assurance of materials are generally based on destructive, semi-destructive, and non-destructive methods [17]. Even though there is limited recognition of ultrasound phenomena in the characterization of liquids, particulates, and porous bodies, several studies [18,19,20,21,22] have emphasized that ultrasonic properties play an important role in material characterization. Moreover, ultrasonic investigations can provide useful information about the microstructural properties, as well as deformation processes in a material, and can predict future performances of the materials. Investigations using ultrasound methods are very precise and much less sensitive to contamination and, therefore, can provide accurate information about the investigated samples. Another important aspect of these techniques is that sound can propagate through various types of samples, such as concentrated, opaque liquid systems, and porous bodies [23,24], offering exceptional insight. Particularly, ultrasound measurements could be used to characterize also concentrated dispersions and emulsions as they are, excluding the need for dilution, which is a requirement in other traditional characterization techniques [23,24,25,26]. In this context, considerable attention has been directed towards the use of ultrasonic methods in material characterization. Ultrasonic investigations are already involved in many fields, such as geology, speleology, oceanography, and medicine. These techniques are becoming of utmost importance in medicine, as both diagnostic tools as well as a therapeutic method. Even though the use of ultrasounds in medicine dates from 1930, it is only quite recently that these techniques have become recognized as important tools in medical practice in ultrasonic imaging, bone evaluation, ophthalmology, intravascular investigations, lithotripsy, hyperthermia, focused ultrasound surgery [27,28,29].

Obtaining nanoscale materials has allowed miniaturization of components, which has led to the possibility of achieving more efficient devices with faster functions and much lower costs. Thus, nanotechnology has come to play a major role in key areas that affect everyday life, such as pharmaceuticals, cosmetics, medical, and environmental applications. This paper aims to provide information on the influence of stability of zinc-doped hydroxyapatite (ZnHAp) colloidal dispersions on the antimicrobial properties of hydroxyapatite doped with various metal ions for researchers conducting studies on finding alternative solutions to the antibiotic used for the treatment of microbial infections.

The present study focuses on the stability and morphology of Zn-doped hydroxyapatite colloidal dispersions, with various zinc concentrations (x_Zn_ = 0; x_Zn_ = 0.07, and x_Zn_ = 0.2). Their morphology and stability were evaluated by SEM, Energy-dispersive X-ray spectroscopy (EDX), Dynamic light scattering (DLS), ζ-potential, and ultrasound measurements. Moreover, the structure of the ZnHAp samples was also evaluated by X-Ray diffraction (XRD) and Fourier-transform infrared spectroscopy (FTIR). Another purpose of this study was to highlight the influence of the stability of the dispersions on the inhibitory effect of ZnHAp against bacterial strains, such as *S. aureus* ATCC 25923 and *E. coli* ATCC 25922.

## 2. Materials and Methods

### 2.1. Sample Preparation

#### 2.1.1. Materials

For the synthesis of ZnHAp, precursors of calcium nitrate (Ca(NO_3_)_2_∙4H_2_O; Sigma Aldrich, St. Louis, MO, USA), ammonium hydrogen phosphate ((NH_4_)_2_HPO_4_; Wako Pure Chemical Industries Ltd., Richmond, VA, USA), Zn(NO_3_)_2_∙6H_2_O (Alpha Aesare, Karlsruhe, Germany; 99.99% purity), ammonium hydroxide (NH_4_OH; Wako Pure Chemical Industries Ltd., Richmond, VA, USA), absolute ethanol (C_2_H_6_O; Sigma Aldrich, St. Louis, MO, USA; ≥99.8% purity), and double distilled water were used.

#### 2.1.2. Zinc-Doped Hydroxyapatite (ZnHAp) Solution

Zinc-doped hydroxyapatite, Ca_10−_*_x_*Zn_x_(PO_4_)_6_(OH)_2_, was prepared by an adapted sol-gel route [30,31] by setting x = 0 (HAp), x = 0.07 (7ZnHAp), and x = 0.2 (20ZnHAp) and (Ca + Zn)/P as 1.67 [15,32]. The synthesis was carried out under atmospheric conditions at a temperature of 100 °C.

In the first step, the (NH_4_)_2_HPO_4_ was dissolved in absolute ethanol using a magnetic stirrer to make P-containing solution. In the second step, the (Ca + Zn)-containing solution was obtained by dissolving Ca(NO_3_)_2_·4H_2_O and Zn(NO_3_)_2_·6H_2_O in a beaker of distilled water under continuous agitation. The P-containing solution was added drop by drop into the (Ca + Zn)-containing solution under continuous agitation at 100 °C. The pH value of the solution was preserved to at 11 by the addition of NH_4_OH. The resulting solution was stirred slowly for 24 h at 100 °C, until the formation of a gel. The aged gels were washed five times using double distilled water and ethanol according to previous studies [30,31,32] and redispersed in double distilled water. The biological and physicochemical properties of the ZnHAp final solution were studied.

### 2.2. Characterization Methods

The morphology of the ZnHAp samples, as well as the chemical composition, was investigated by scanning electron microscopy (SEM) using a HITACHI S4500 (Hitachi, Ltd., Chiyoda, Japan) microscope equipped with an EDX attachment operating at 20 kV.

The structure of the ZnHAp samples was investigated by XRD measurements using a Bruker D8 Advance diffractometer (Bruker, Karlsruhe, Germany) with a nickel-filtered Cu Kα (λ = 1.5418 Å) radiation in the 2θ ranging from 20° to 80°. Furthermore, the functional groups of the prepared samples were identified using FTIR spectroscopy with a Perkin Elmer Spectrum BX spectrometer (Waltham, MA, USA).

DLS analysis and ζ-potential were performed at 25 ± 1 °C using an SZ-100 Nanoparticle Analyzer (Horiba, Ltd., Kyoto, Japan). All samples were diluted in double distilled water 10 times before measurements of DLS and ζ-potential. The dynamic light scattering (DLS) technique is based on Rayleigh scattering from suspended nanoparticles that are subject to Brownian motion. The hydrodynamic diameter of the nanoparticles can be determined by illuminating the sample with a laser source that allows us to appreciate the particle diffusion velocity. To record the scattered signals, ZnHAp solutions were placed in the disposable cuvettes. For each analyzed sample, three determinations were recorded. The final value was established by mediating the three measurements.

**Figure 1:**
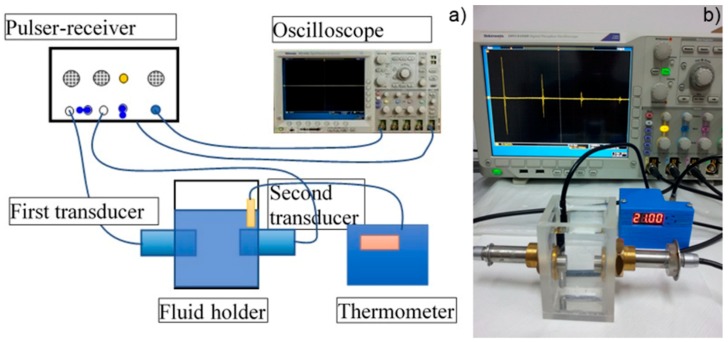
Experimental setup for ultrasound measurements. Schematics (**a)** and image (**b**) [7].

The ultrasound studies took place in a specialized laboratory, using two identical ultrasonic transducers H5K (General-Electric, Krautkramer, Hürth, Germany) of 5 MHz central frequency as previously reported in Predoi et al. [7]. The experimental set-up is depicted in Figure 1.

In order to quantify the zinc present in the samples, flame atomic absorption spectrometry (AAS) studies were conducted on the ZnHAp solutions. For this purpose, AAS studies have been performed using a Zeeman HITACHI Z-8100 from Japan Hitachi (Tokyo, Japan) on stable solutions of ZnHAp. The experiments were performed in triplicate.

### 2.3. Antimicrobial Assays on Staphylococcus Aureus and Escherichia Coli Strain

The quantitative assay regarding the antimicrobial effect of ZnHAp colloidal dispersions (x_Zn_ = 0, x_Zn_ = 0.07, and x_Zn_ = 0.2) was done using adapted two-fold serial dilutions, previously described in [15]. The bacterial strains used in the antimicrobial studies were *Staphylococcus aureus* ATCC 25923 and *Escherichia coli* ATCC 25922.

## 3. Results and Discussions

The purpose of this study was to analyze the influence of zinc substitution for calcium from hydroxyapatite on the morphology and colloidal stability of ZnHAp nanoparticles in aqueous solutions. In previously reported studies by J. Lyklema, colloid was defined as “an entity” having at least one direction, and a dimension between 1 nm and 1 mm [33] has been taken into account. According to the definition given by J. Lyklema, these entities may be solid, liquid or gaseous, with a wide variety of systems meeting this broad definition of colloids. In our study, the dispersal medium was water. For the first time, particular attention was paid to the influence of ZnHAp nanoparticles in aqueous solutions on the development of bacteria, such as *E. coli* and *S. aureus*.

**Figure 2:**
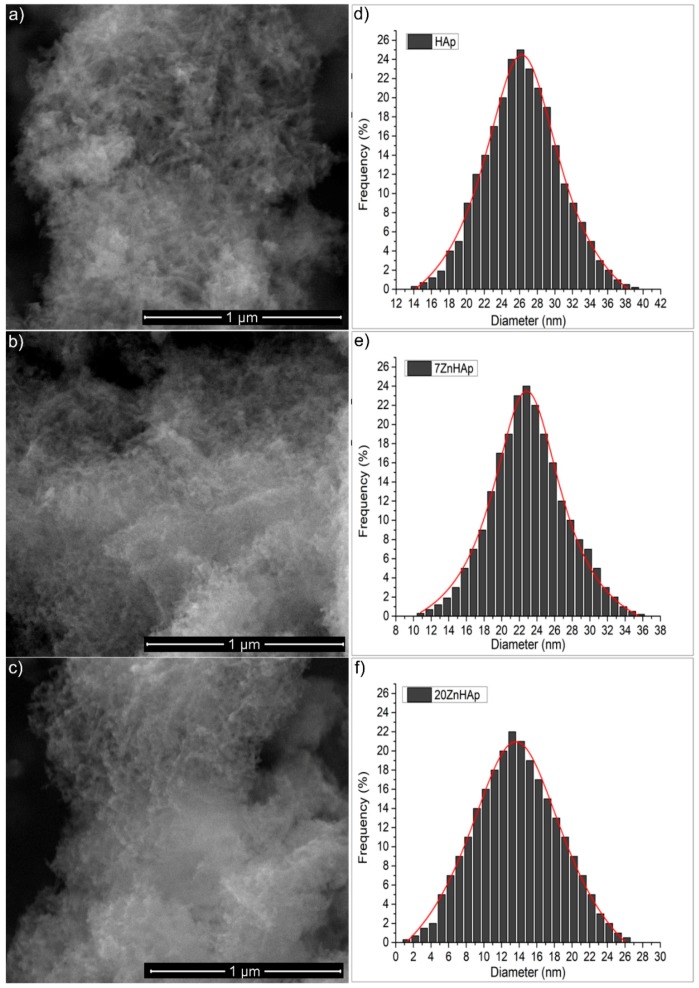
The SEM images of hydroxyapatite (HAp) (**a**) 7ZnHAp (**b**), and 20ZnHAp (**c**) and their corresponding particle size distribution (**d**–**f**). ZnHAp: zinc-doped hydroxyapatite.

The morphological study of the ZnHAp samples with different Zn concentrations was performed using the electronic scanning microscope, while the EDX analysis was used to determine the elemental composition of the ZnHAp samples. The morphology and size of the ZnHAp (with different Zn^2+^ concentrations) samples were revealed from the high-resolution SEM micrographs. The SEM images recorded with a magnification of ×100,000, 30 kV (a spot of 3.5), and the particle size distribution is shown in Figure 2. The images of the prepared ZnHAp nanoparticles (Figure 2a–c) suggested that the substitution of Zn^2+^ in HAp did not produce significant changes in HAp morphology. All the powders (HAp, 7ZnHAp, and 20ZnHAp) were made up of nanoparticles with elongated morphology. Average particle diameter (D_SEM_) deduced from the particle size distribution of ZnHAp samples (Figure 2d–f) decreased as the zinc concentration increased. The D_SEM_ for HAp samples was 26.2 ± 0.1 nm, while the D_SEM_ for 7ZnHAp and 20ZnHAp dropped from 22.8 ± 0.2 to 13.6 ± 0.2 nm, respectively.

**Figure 3:**
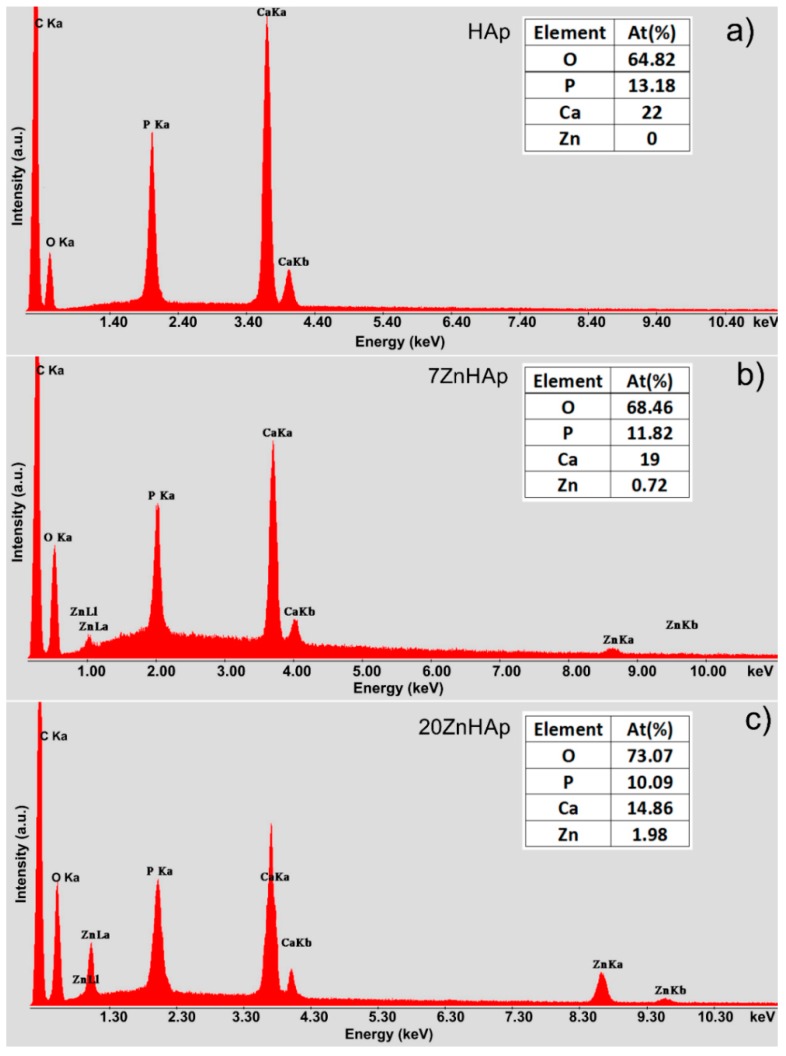
Energy-dispersive X-ray spectroscopy (EDX) spectra of hydroxyapatite (HAp) (**a**), 7ZnHAp (**b**), and 20ZnHAp (**c**) samples. ZnHAp: zinc-doped hydroxyapatite.

EDX analysis was accomplished for the ZnHAp (in order to establish the elemental composition (Figure 3). In Figure 3a–c, the EDX analysis of HAp, 7ZnHAp, and 20ZnHAp are presented. The spectra of 7ZnHAp and 20ZnHAp showed the characteristic peaks of Zn, Ca, P, and O, while in the HAp spectrum, only the characteristic peaks associated with O and P were evidenced. The results of EDX analysis of HAp, 7ZnHAp, 20ZnHAp samples are presented in Table inserted in Figure 3. The EDX analysis indicated that the intensity of Ca decreased when the intensity of zinc increased as the zinc content was increased in the hydroxyapatite structure. According to previous studies [34], the decreasing of Ca intensity as Zn content increases may be due to the fact that CaO has been substituted by ZnO [34].

**Figure 4:**
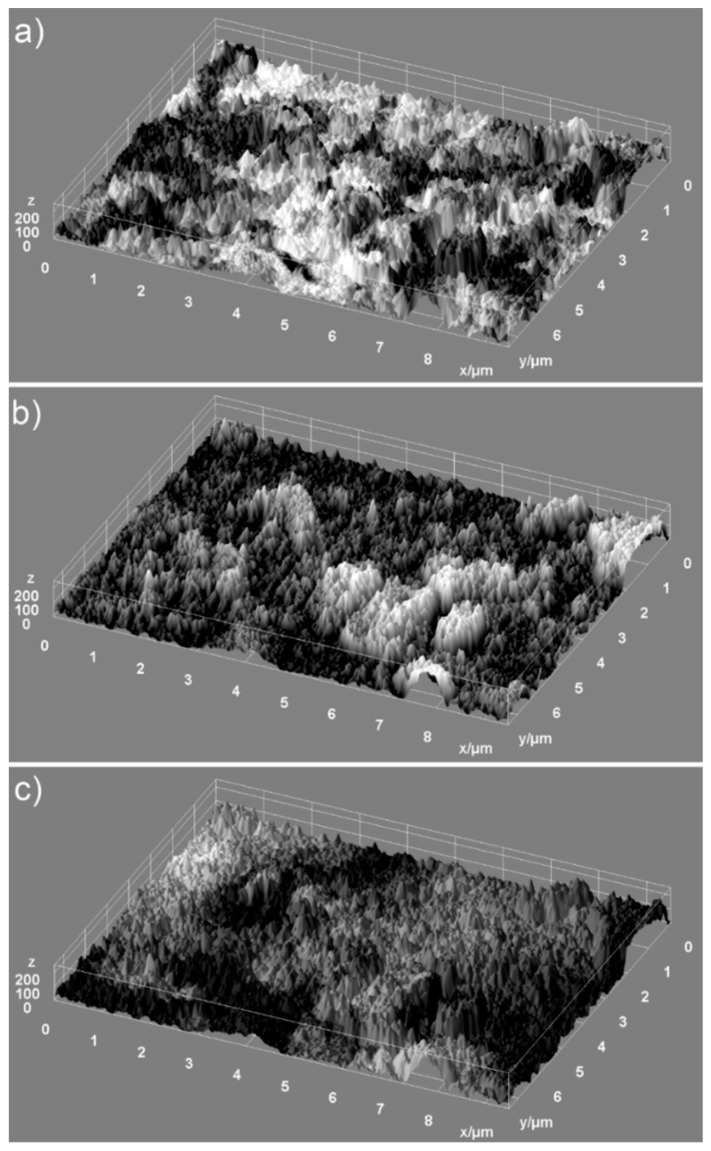
3D surface of SEM images of the selected area for elemental analysis of hydroxyapatite (HAp) (**a**) 7ZnHAp (**b**), and 20ZnHAp (**c**) samples. ZnHAp: zinc-doped hydroxyapatite.

Information regarding the uniformity of the constituents of the analyzed samples and the homogeneity of the samples were provided by the elemental mapping analysis. 2D conventional images can be transformed into 3D images for accurate morphology assessments of the samples using special software programs [35,36]. Therefore, in our study, we have used 2D SEM selected images in order to obtain 3D surface maps of the ZnHAp dispersions for elemental cartographic analysis. The 3D surface maps were realized starting from the selected area of SEM images of the samples (HAp, 7ZnHAp, and 20ZnHAp) of approximately 9.5 µm × 7.5 µm in dimension using Image J software [37]. The 3D representation selected areas of SEM images of the HAp, 7ZnHAp, and 20ZnHAp powders surface morphology are presented in Figure 4. Furthermore, the 3D SEM surface topographies suggested that the particles present the tendency to agglomerate and that the particles show a slight decrease in size when Zn concentration increases (Figure 4a–c).

**Figure 5:**
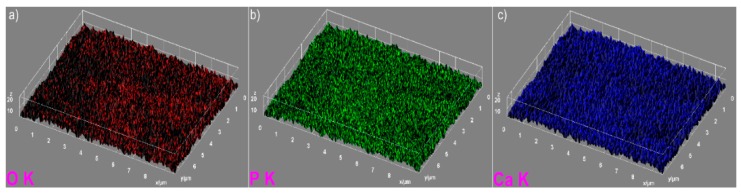
The 3D images of elemental mapping analysis of the hydroxyapatite (HAp) samples, oxygen (**a**), phosphorus (**b**), calcium (**c**).

**Figure 6:**
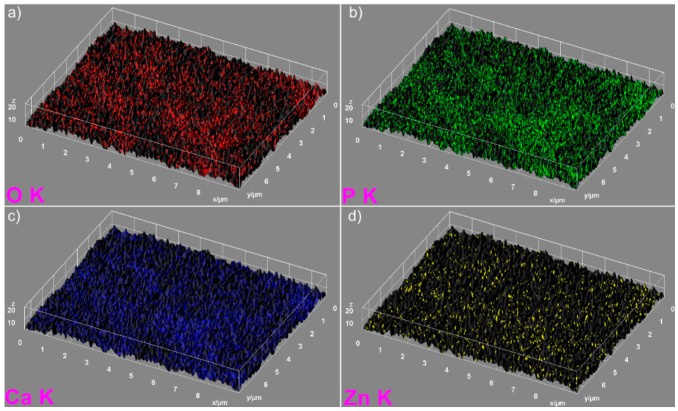
3D images of elemental mapping analysis of the 7ZnHAp samples oxygen (**a**), phosphorus (**b**), calcium (**c**) and zinc (**d**). ZnHAp: zinc-doped hydroxyapatite.

**Figure 7:**
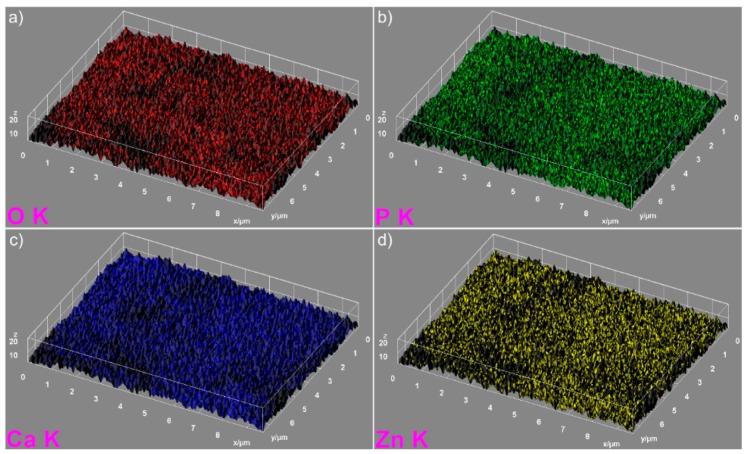
The 3D images of elemental mapping analysis of the 20ZnHAp samples. oxygen (**a**), phosphorus (**b**), calcium (**c**) and zinc (**d**) ZnHAp: zinc-doped hydroxyapatite.

The elemental mapping analysis provided information on both the uniform distribution of the elements sample and the homogeneity of the samples. The 3D topographies of the EDX elemental mapping analysis obtained using Image J software of the HAp, 7ZnHAp, and 20ZnHAp samples are presented in Figure 5, Figure 6 and Figure 7. In Figure 5, from the elemental mapping represented in 3D, it can be clearly seen that the main constituents of hydroxyapatite, O, Ca, and P were uniformly distributed in the sample. The 3D representation of the elemental mapping revealed that the HAp powder is composed of oxygen, phosphorus, and calcium, the main constituents of hydroxyapatite. The uniform distribution of Zn, Ca, P, and O was also observed (Figure 6 and Figure 7) in the 3D topographies of the elemental maps of the 7ZnHAp and 20ZnHAp samples. More than that, the 3D surface images evidenced that the distribution of O(K), P(K), Ca(K), and Zn(K) elements in the ZnHAp was homogenous and uniform. All the SEM analysis results obtained in this study were in good agreement with previously reported studies on ZnHAp powders [14,38]. Furthermore, in order to assess the quantity of zinc from the ZnHAp samples, flame atomic absorption spectrometry studies were conducted on the stable solutions of 7ZnHAp and 20ZnHAp. The results of the AAS investigation revealed that the measured zinc concentrations from the samples 7ZnHAp and 20ZnHAp were 0.996 ± 2.7 wt.% and 2.375 ± 3.6 wt.%, respectively. In agreement with previous studies [39], slightly different values obtained for the molar ratio, (Ca + Zn)/P, following AAS and EDX studies could suggest that this ratio was lower on the surface of the particles than inside them. Moreover, studies on surface characterization of hydroxyapatite related calcium phosphate [40] showed that the powders of the analyzed samples had surface stoichiometries similar to their bulk crystal compositions when the composition of the surface represented 1–10% of the bulk.

**Figure 8:**
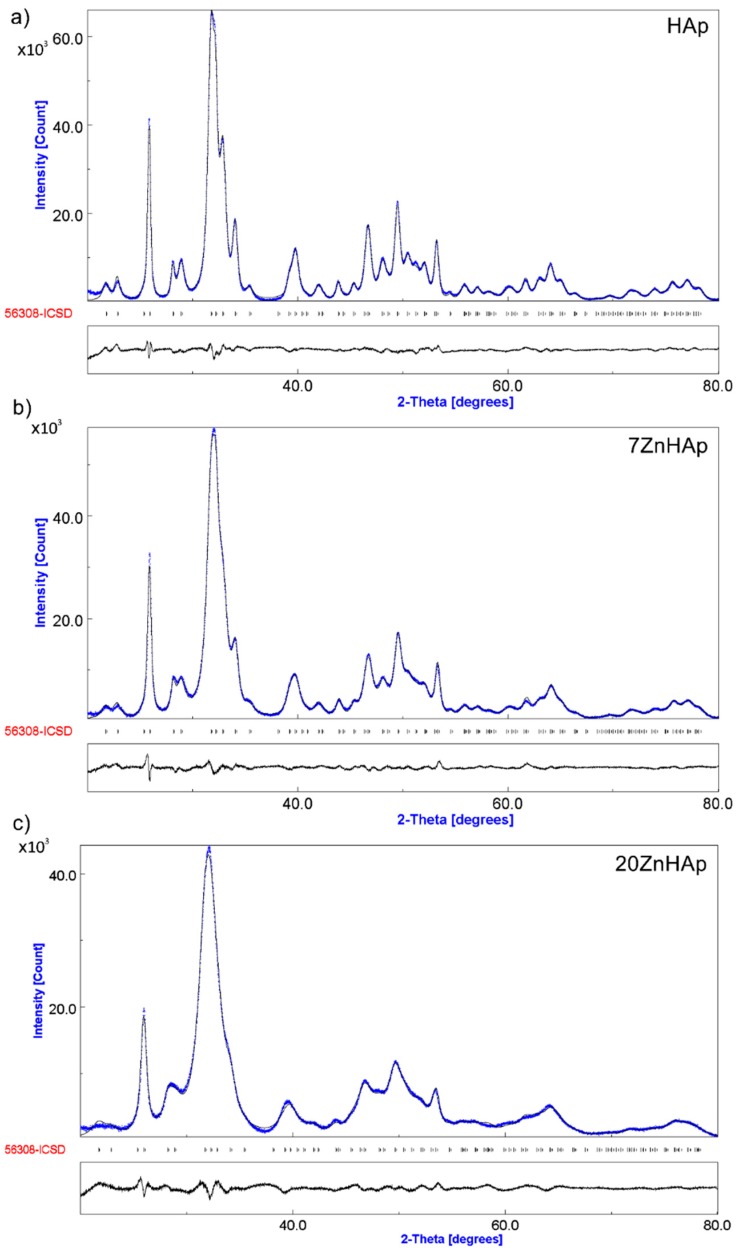
Rietveld refinement fit of powder XRD data of hydroxyapatite (HAp) (**a**), 7ZnHAp (**b**), and 20ZnHAp (**c**). The blue line marks the experimentally observed pattern, while the gray line marks the calculated diffraction pattern. The positions of the calculated Bragg peaks were marked with the vertical lines. The difference pattern between observed and calculated patterns was represented by the gray line at the bottom of the figure. ZnHAp: zinc-doped hydroxyapatite.

In Figure 8, both the experimental data (marked in blue) and the calculated data (gray line) obtained by Rietveld refining of the obtained samples are presented. The Rietveld refinement was achieved using MAUD (Material Analysis Using Diffraction) program [41].

The positions of the diffraction lines of the hexagonal hydroxyapatite (ICDD-PDF No. 9-432) are represented by the vertical lines. The difference between experimental data and those calculated is represented by the gray line at the bottom of the figure. It was noted that between the experimental data and the calculated data, a good similarity was observed. Following the use of the Rietveld refining method for the XRD analysis of the obtained samples, a single phase corresponding to the hexagonal hydroxyapatite was revealed. The calculated lattice parameters of HAp, 7ZnHAp, and 20ZnHAp samples are in good accord with the standard data of a = b = 9.418 Å, c = 6.884 Å. For the HAp sample, the values of a = b and c were 9.4320 Å and 6.8838 Å, respectively. The calculated lattice parameters of 7ZnHAp sample were a = b = 9.4362 Å and c = 6.8806 Å. For 20ZnHAp sample, the calculated values of lattice parameters were a = b = 9.4387 Å and c= 6.8736 Å. The average crystallite sizes decreased with the increase of zinc concentration from 23.18 ± 0.3 nm for HAp sample to 19.38 ± 0.5 nm and 9.96 ± 0.8 nm for 7ZnHAp and 20ZnHAp sample, respectively. For the analyzed samples, an increase in the network parameter “a” was observed with increasing zinc concentration in the sample, while for parameter “c” a decrease was observed.

The correctness of the Rietveld refinement of the obtained samples is monitored by a number of parameters, such as the index of the weighted profile *R*_wp_ and the index χ. The χ index is given by the ratio of statistically estimated *R*_wp_ and *R*_exp_ factors and represents the “correctness of the overlap between experimental and calculated data”. On the other hand, the R_Bragg_ factor that can be determined using the Rietveld method for data processing is very useful because its value depends on the fit of the structural parameters. The values obtained for *R*_wp_ factor were 2.412 for HAp, 2.329 for 7ZnHAp, and 2.220 for 20ZnHAp. For HAp, 7ZnHAp, and 20ZnHAp samples, the values obtained for *R*_exp_ factor were 0.934, 0.918, and 0.900. The values of R_Bragg_ factor were 1.847 for HAp and 1.752 for 7ZnHAp, while for 20ZnHAp, it was 1.7692. The theoretical values of the factors *R* obtained for all ZnHAp samples are consistent with Toby’s theory [42]. Moreover, the processing of the diffraction spectrum of the samples has shown that the analyzed samples exhibit the characteristics of the hexagonal hydroxyapatite without revealing another supplemented phase and having a good crystallinity. Our results were confirmed by previous studies on the formation and structure of zinc-substituted calcium hydroxyapatite that have shown that crystallinity decreases when the Zn concentration increases [43].

The results we have identified, in this study, are confirmed by previous studies by A. Bigi et al. [44] in their research on the inhibitory effect of zinc on the crystallization of hydroxyapatite. On the other hand, the same authors [44] have missed that both the synthesis method and the pH at which the synthesis is performed can influence the values of the lattice parameters “a” and “c”. Furthermore, a major role in obtaining a single phase corresponding to hexagonal hydroxyapatite is played by synthesis parameters, the temperature being one of the most important. pH also plays a very important role. More of that, the time at which the two solutions are mixed, and the temperature at which the mixture takes place, also play an important role in obtaining a stable structure characteristic of the hexagonal hydroxyapatite. Z. Salima et al. [45] in studies on characterization of magnesium-doped hydroxyapatite prepared by sol-gel process reported that all powders are composed of pure apatite phase even after heat treatment at 500 °C. S. Kannan et al. [46,47] also reported the presence of beta-tricalcium phosphate phase. Wilcock et al. [48] in their studies on silver-doped hydroxyapatite reported the presence of additional phases. Moreover, in the calcined samples at 1200 °C, with an increased amount of silver doping [48], a greater amount of β-tricalcium phosphate (β-TCP) was detected. On the other hand, studies conducted by C.L. Popa et al. [49] suggested that the structure of the silver-doped hydroxyapatite (x_Ag_ = 0.5) changes gradually, from hydroxyapatite (sample dried at 40 °C) to a predominant β-TCP structure achieved when the thermal treatment temperature was 1000 °C. C. S. Ciobanu et al. [50] in their studies regarding the influence of annealing treatment on the bioceramics properties showed that the hydroxyapatite structure did not change after heat treatment at 600 °C. Furthermore, it has been found that with the rise in temperature, the peaks of hydroxyapatite were sharpened, and at temperatures of 800 °C, a weak peak of calcium oxide (CaO) appeared, and the concentration of calcium oxide increased after thermal treatment at 1000 °C [50]. M.F. Hsieh et al. [51], showed that the occurrence of CaO at high temperatures (≥800 °C) might be due to the chemical decomposition of the remaining calcium nitrate. Both the temperature in the first stage of the synthesis process and the pH during the synthesis process could influence the stability of the hydroxyapatite structure and could lead to the presence of β-TCP.

**Figure 9:**
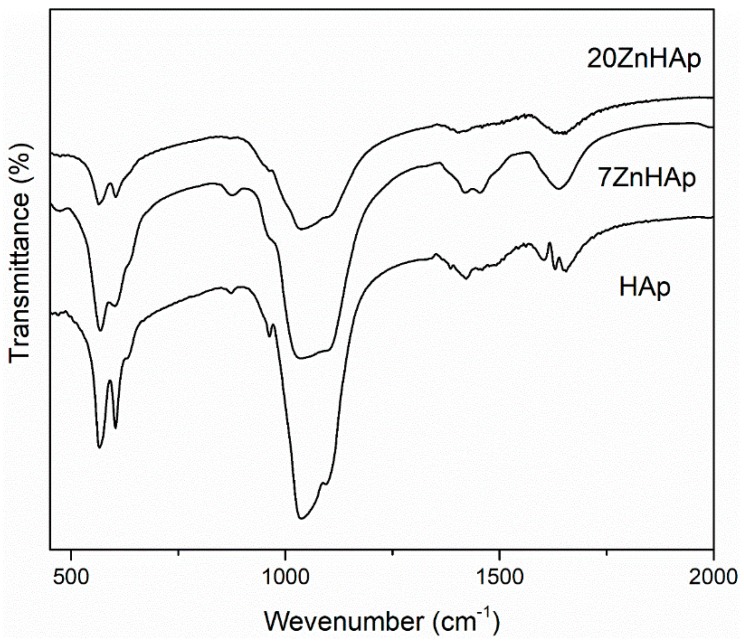
FTIR spectra for the different Zn-doped hydroxyapatite.

In Figure 9, the FTIR spectra for prepared samples are shown. According to previous studies [46,52], the bands located at around 604 and 563 cm^−1^ represent the triply degenerated bending modes of the O–P‒O bond (ν_4_), while the band at 960 cm^−1^ can be attributed to the symmetric stretching mode of the P–O bond (ν_1_). The bands localized at around 1095, 1033 indicated the presence of PO_4_^3−^ group [47]. In the spectral range 870–880 cm^−1^, the band attributed to the HPO_4_^2−^ was found. The bands in the spectral range 1414–1450 cm^−1^ could be attributed to the carbonate functional group [53]. The presence of bands describing C-O vibrations suggests that when synthesis is carried out at low temperatures, a certain amount of carbonate has been incorporated into samples [53]. The spectrum presented in Figure 9 also indicates the existence of H_2_O (1641 cm^−1^). The FTIR spectra of the analyzed samples show a widening of the peaks and a smoothing as the zinc concentration increases in the sample. This behavior could suggest a decrease in the crystallinity of the samples with increasing zinc concentration. These results are consistent with previous experimental results [54] which showed that crystal size and crystallinity decreased when Zn concentration increased.

**Figure 10:**
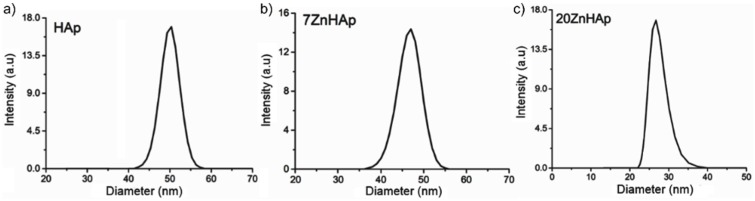
Dynamic light scattering (DLS) particle size distribution curve of hydroxyapatite (HAp) (**a**), 7ZnHAp (**b**) and 20ZnHAp (**c**) nanoparticles. ZnHAp: zinc-doped hydroxyapatite.

In order to calculate the hydrodynamic diameter, the Stokes-Einstein equation [55] was used. Particle size distribution of HAp, 7ZnHAp, and 20ZnHAp particles in solution determined by DLS method is presented in Figure 10. The average hydrodynamic diameter of the particles (D_HYD_) achieved for HAp by DLS was 51.8 ± 0.3 nm, while for 7ZnHAp and 20ZnHAp, it was 47.5 ± 0.3 and 26.81 ± 0.4 nm, respectively. The results of DLS studies accomplished on prepared samples revealed that the average hydrodynamic diameter of the particles decreased when Zn concentration increased in agreement with structural analysis.

A decrease in nanoparticle size was established by both SEM studies and DLS measurements. The decrease in particle size could be attributed to the increase of Zn content incorporated in HAp [11,14,56]. The difference could be explained by the fact that the SEM method determines the diameter of the metallic core of the particles as the shell representing the coating of the metallic core is destroyed over the drying and in the vacuum chamber of the SEM. The hydrodynamic particle size calculated from DLS technique is given by the metallic core of the particles, the adsorbed substances on their surface, and the thickness of the electrical double layer that moves along with the particle, which leads to a larger particle size [57].

**Figure 11:**
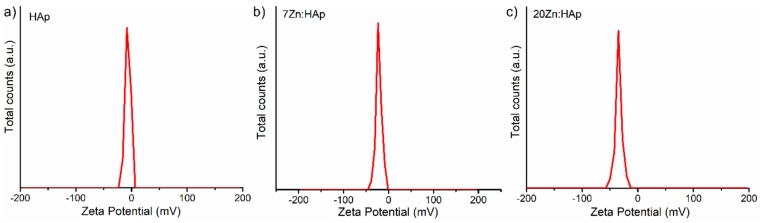
The behavior of the ζ-potential of hydroxyapatite (HAp) (**a**), 7ZnHAp (**b**), and 20ZnHAp (**c**) particle suspensions. ZnHAp: zinc-doped hydroxyapatite.

In addition, the ζ-potential of the HAp, 7ZnHAp, and 20ZnHAp aqueous solutions were appraised. The ζ-potential and ultrasound measurements can allow us to obtain information about the stability of a colloidal system. It is well known that the ζ-potential is one of the key parameters that give us information on the stability of colloidal dispersions. In agreement with the previously presented studies [58,59], colloidal solutions with high potential zeta were electrically stable (the net electrical charge on the surface of the particles is higher and thus the electrostatic repulsion between the particles). On the other hand, colloidal solutions that have a low ζ-potential have a tendency to coagulate or flocculate. The determined ζ-potential value of HAp, 7ZnHAp, and 20ZnHAp was −7.83 mV, −23.16 mV, and −34.65 mV, respectively (Figure 11).

For HAp (x_Zn_ = 0), the value of ζ-potential is lower than −30 mV. When the zinc concentration increases in the sample, ζ-potential increases reaching a value of −34.65 mV for 20ZnHAp (x_Zn_ = 0.2), the system arrives in a state of moderate stability. The value of ζ-potential for HAp is in agreement with the results obtained previously. The value of ζ-potential for pure HAp reported by Liu et al. [60] was −5 mV, while Wahba et al. [61] showed that ζ-potential of pure HAp was −9 mV, and the value increased for cerium-doped hydroxyapatite to −20 mV. Predoi et al. [62] also reported a value of ζ-potential equal to −8.19 mV for zinc-doped hydroxyapatite (x_Zn_ = 0.01) synthesized by an adapted co-precipitation method.

**Figure 12:**
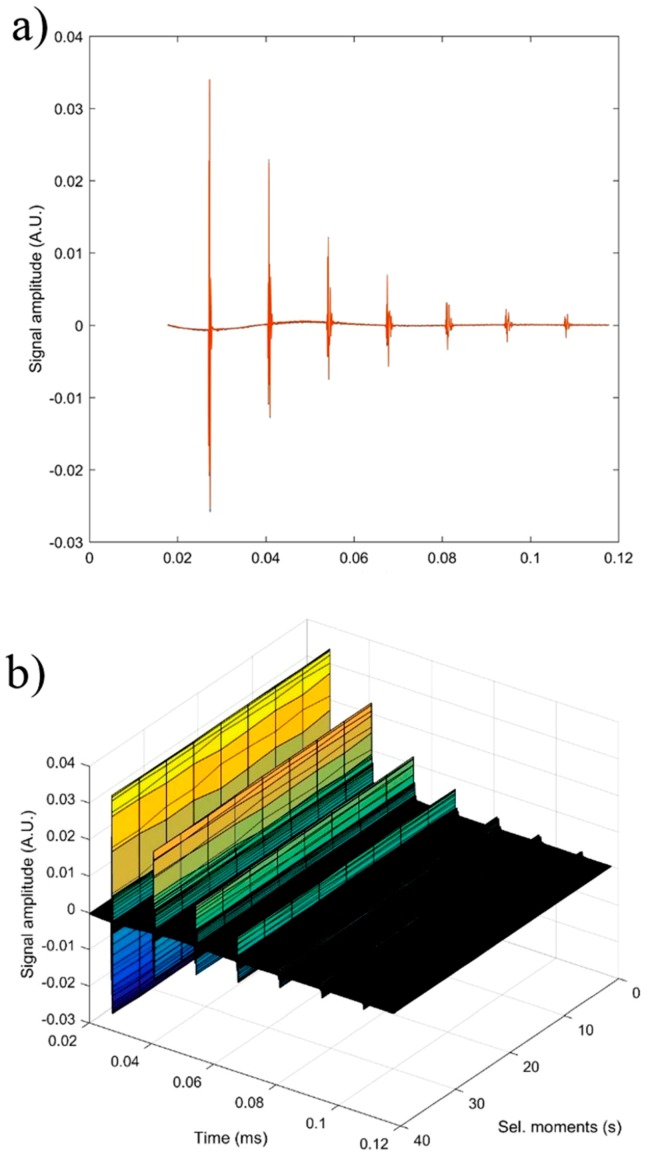
Signals recorded for water during a 40 s monitoring: seven recorded echoes, repeating the recording every 5 s (**a**) and unchanged fluid properties (**b**).

**Figure 13:**
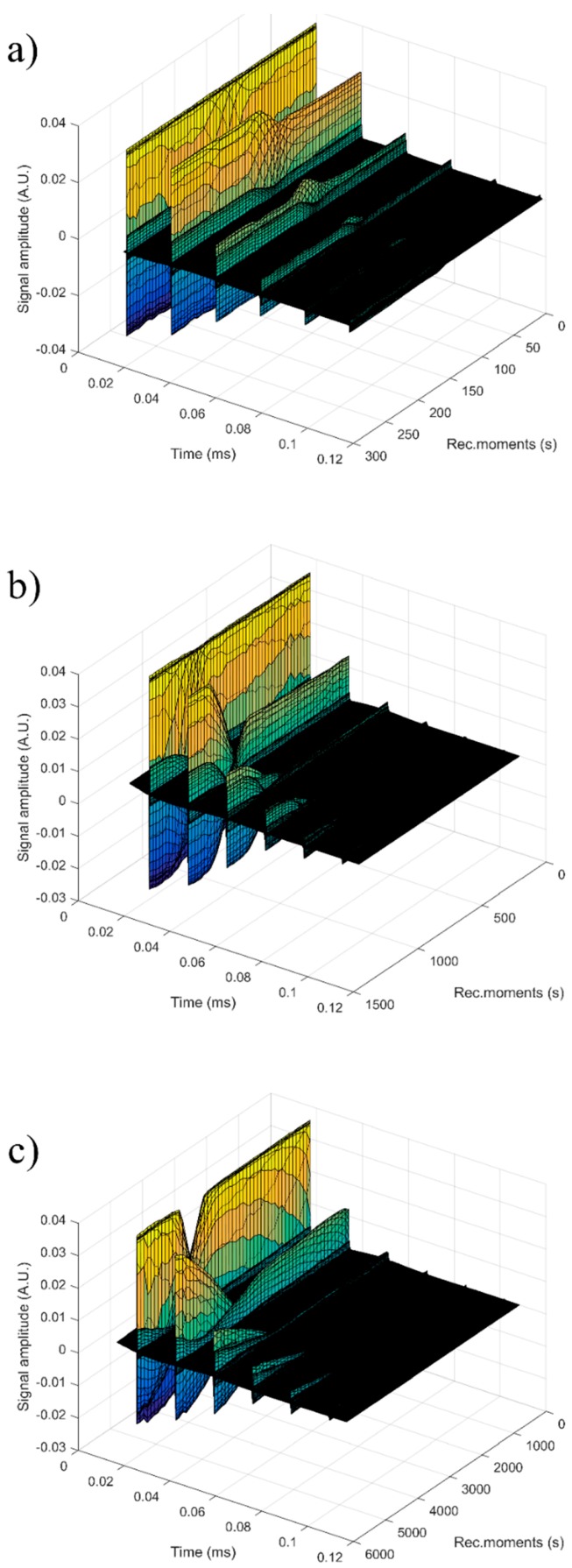
Signals recorded for samples hydroxyapatite (HAp) (**a**), 7ZnHAp (**b**), and 20ZnHAp (**c**). ZnHAp: zinc-doped hydroxyapatite.

In order to obtain supplementary information about the ZnHAp (x_Zn_ = 0, x_Zn_ = 0.07, and x_Zn_ = 0.2) particle suspensions, ultrasound measurements were performed. Ultrasound measurements are a major advantage compared to other techniques, such as DLS or ζ-potential, as ultrasounds can propagate through concentrated suspensions, allowing characterization of concentrated dispersions without dilution. Dilution of suspensions for DLS or ζ-potential measurements can destroy aggregates or flocculation, which could lead to unclear information. The ultrasonic signals have been recorded every 5 s, and 5 to 7 echoes were recorded. The number of echoes recorded during this interval was dependent on the signal-to-noise ratio. The first recorded signal was the direct signal between the first transducer and the second transducer. We can mention here that the first transducer is the signal generator, while the second transducer is just a receiver. The rest of the signals are the consecutive echoes identified by the two transducers, as the ultrasonic chirp is reflected by the transducers circular surfaces and traveling along the distance between these surfaces. The distance between transducers was selected d = 25 mm. The reference fluid is double distilled water (H_2_O). In Figure 12a seven recorded echoes, repeating the recording every 5 s, for an experiment which lasted 40 s are shown. As expected, the signals were identical since, during the 40 s, the fluid properties remained unchanged (Figure 12b). For samples HAp (x_Zn_ = 0) to 20ZnHAp (x_Zn_ = 0.2), the recorded signals are shown in Figure 13.

**Figure 14:**
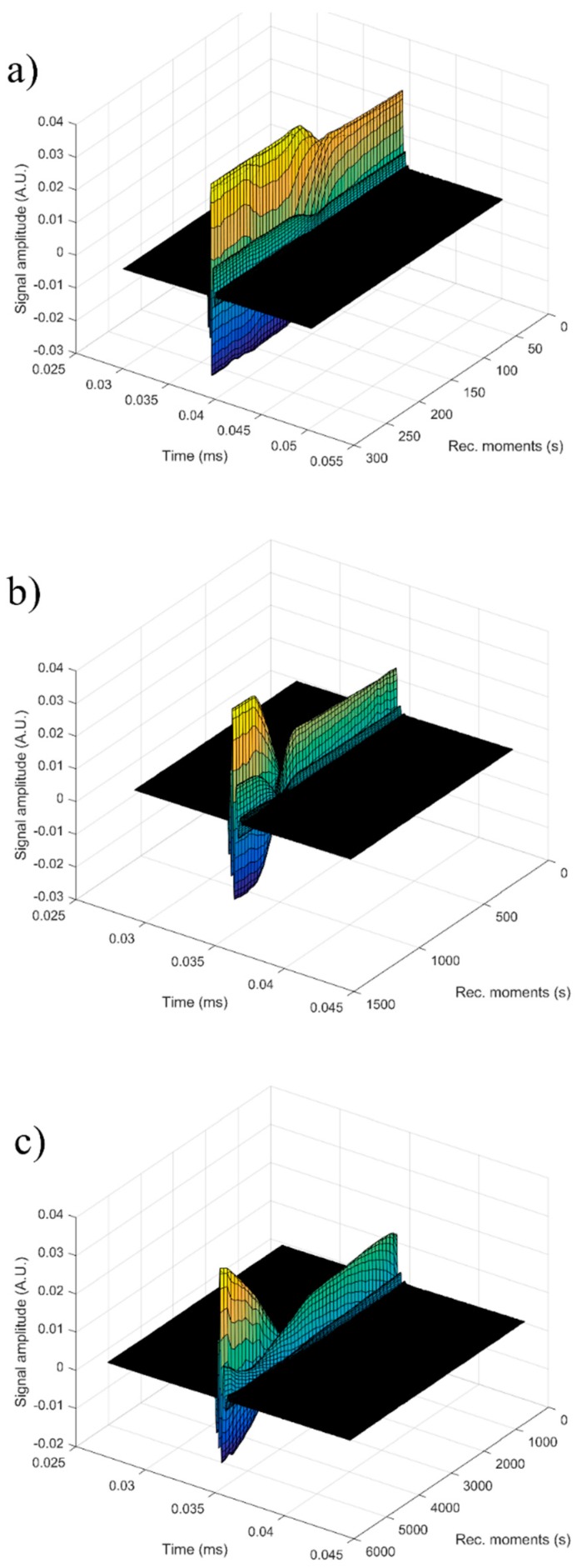
The same 2nd echo of hydroxyapatite (HAp) (**a**), 7ZnHAp (**b**), and 20ZnHAp (**c**) samples selected for investigation. ZnHAp: zinc-doped hydroxyapatite.

For each sample, the first recorded chirp that represents the direct signal, arriving after around 0.017 ms, was amplified up to the saturation of the amplifier. The purpose was to record five echoes even for the most attenuative sample. For this reason, the investigation was focused on the second echo, which traveled twice the distance between the transducers and was thus more attenuated. In Figure 14, the selected echoes for the three samples are shown. Apparently, a sample exhibits different behavior from the other. Sample HAp exhibits a continuous increase of signal amplitude at three stages (i) bulk precipitation lasting 110 s for HAp in this experiment, (ii) transition of the separation surface in front of the transducers (from 110 s to 150 s in this experiment), (iii) slow precipitation of remaining particles behind the separation surface, during which the amplitude of the signal increases slowly, up to the level reached in the pure solvent (H_2_O).

Samples 7ZnHAp and 20ZnHAp were selected after (a) an initial stage during which the signal amplitude varies slowly and (b) the reach stage during which there is a drop in signal amplitude. This behavior could be due to particles concentration in front of the advancing separation surface and also the variation of the acoustic wave velocity. It was remarked as a significant variation of the acoustic wave velocity in the suspension and in the solvent. It was also found that the different record time was used for the three samples: from 300 s (H_2_O) to 1500 s (HAp) and 5500 s (7ZnHAp).

The most significant parameter, defining the suspension stability, is the amplitude variation during the initial stage (a). It is necessary to mention that this variation can be an increase or a decrease of amplitude, due to acoustic waves dispersion.

Consequently, we can say that stability can be quantified by the absolute value of the amplitude variation during the initial stage. It has been observed that both the transducer generated signal amplitude and the received signal amplification are influencing the measured amplitude of the signal. As a result, the slope of the amplitude-time function was normalized by the average amplitude during the stage (a). In this study, the stability parameter will be calculated according to the equation:

(1) $$s=\frac{1}{A_{m}}\left|\frac{dA}{dt}\right|$$

**Figure 15:**
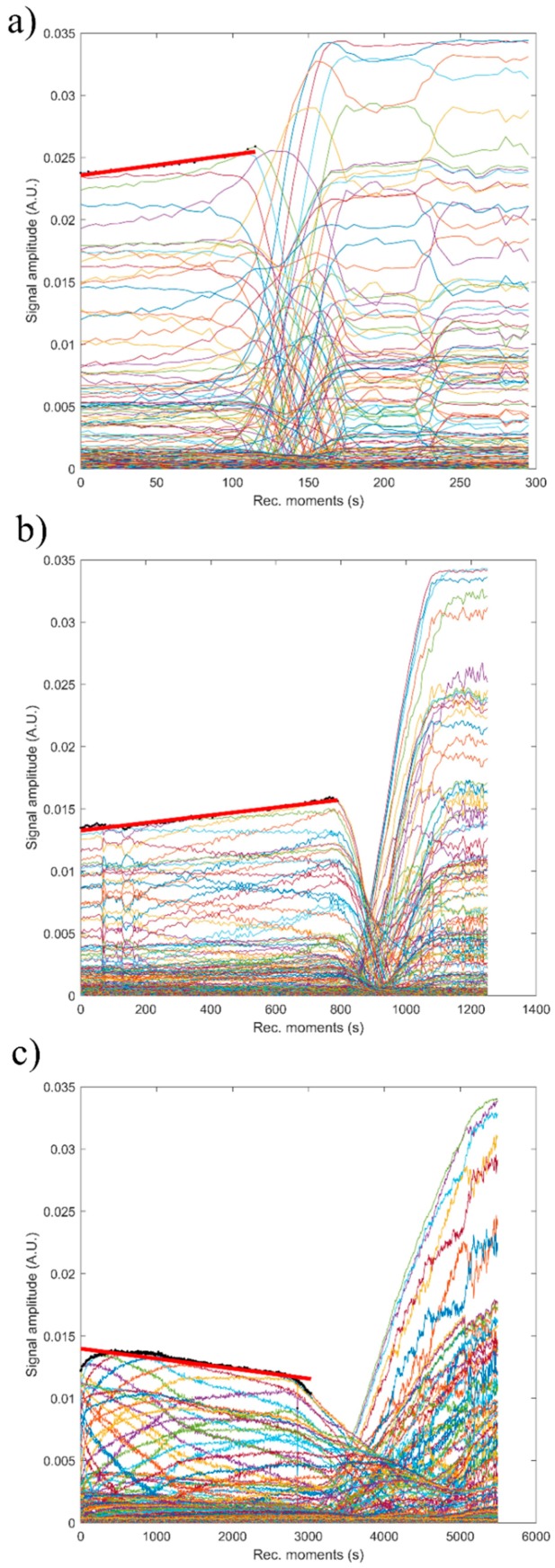
Evolution in time of the amplitudes of the selected echo, for the samples hydroxyapatite (HAp) (**a**), 7ZnHAp (**b**), and 20ZnHAp (**c**). ZnHAp: zinc-doped hydroxyapatite.

Figure 15 shows the absolute value of amplitudes due to the second echo for the three studied samples. The red line revealed the linear best fit, calculated using the proposed method, to calculate the stability parameter. It can be seen that stability over time was different from one sample to another. This behavior shows that the stability of the studied samples is strongly influenced by zinc concentration.

The reference fluid (H_2_O), after taking sufficient samples, is proven to be stable as expected (s = 0). The stability parameter has decreased from 0.00000 ± 1 × 10^−6^ in reference fluid to 0.00066 s^−1^ in sample HAp. Moderate stability was observed in sample 7ZnHAp when the stability parameter was equal to 0.00021 s^−1^. A very stable parameter of only 0.00007 s^−1^ was determined for sample 20ZnHAp. The sample 20ZnHAp has a short initial period when the amplitude increases, followed by a linear decrease of amplitude, attributed to the fluid turbulence after the intense stirring preceding the experiment.

**Figure 16:**
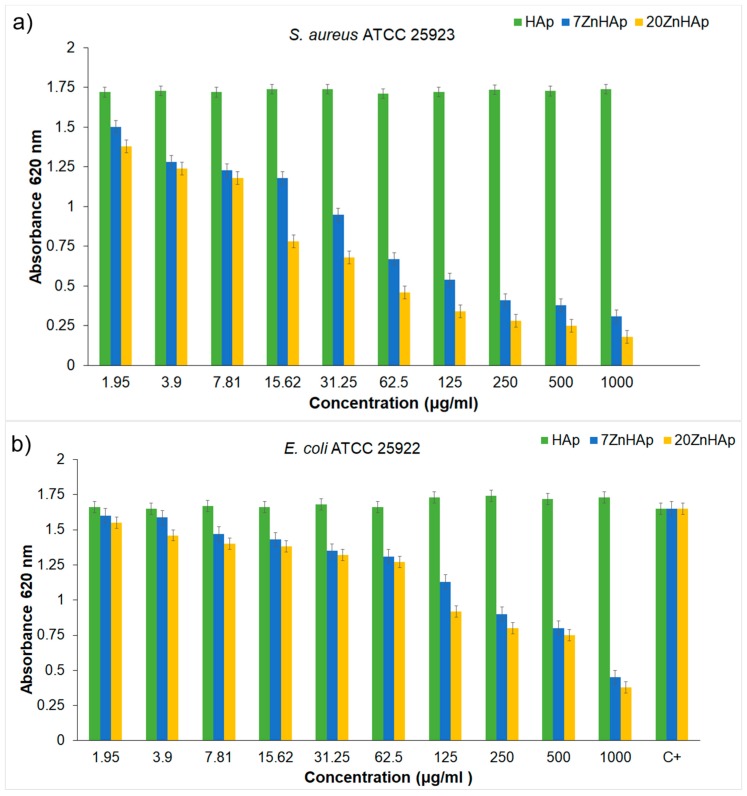
*S. aureus* ATCC 25923 (**a**) and *E. coli* ATCC 25922 (**b**) cell growth in LB at 30 °C for 12 h in the presence of zin-doped hydroxyapatite (ZnHAp) with x_Zn_ = 0, x_Zn_ = 0.07 or x_Zn_ = 0.2 at concentrations between 1.95 and 1000 µg/mL.

As a conclusion of these experimental techniques, we mention the possibility to assess the suspension stability from ultrasonic amplitude variation in time. During the first stage, the amplitudes showed a linear variation in time, so that a relatively short duration of the experiment (1–2 min) is sufficient to determine the stability parameter. The results obtained by ultrasound-based technique realized on ZnHAp final solution resulted after synthesis (concentrated dispersions without any dilution) confirmed the stability of the tested solutions revealed by the traditional ζ-potential characterization method. Establishing the stability of solutions obtained as such without dilution is very important because the dilutions required to use traditional ζ-potential method can destroy the aggregates and the dilution changes the suspension medium. Results obtained in this study revealed that the stability of nanoparticles was influenced by the Zn/(Zn + Ca) ion ratios. It has been found that the stability of ZnHAp solutions increases with the increase in zinc content. ZnHAp is of major medical interest since the presence of hydroxyapatite and Zn was revealed in biological tissues, such as bone and enamel of human teeth [62]. Moreover, it has been shown that besides the biological properties, Zn-doped hydroxyapatite has an inhibitory effect on the development of bacteria and pathogenic yeasts and fungi, such as *E. coli*, *S. aureus*, *Candida albicans,* and *Streptococcus mutans* [63]. In our study, we wanted to highlight, for the first time, the influence of zinc-doped hydroxyapatite dispersions on the development of bacteria, such as *S. aureus* ATCC 25923 and *E. coli* ATCC 25922. The antibacterial activity of ZnHAp colloidal dispersions (x_Zn_ = 0, x_Zn_ = 0.07, and x_Zn_ = 0.2) at concentrations ranging between 1000 and 1.95 μg/mL against *S. aureus* ATCC 25923 and *E. coli* ATCC 25922 were investigated. *S. aureus* ATCC 25923 and *E. coli* ATCC 25922 cell growth in LB at 30 °C for 12 h in the presence of the tested compounds at various concentrations are presented in Figure 16a,b. The biocidal effect of the 7ZnHAp and 20ZnHAp samples was observed on the two studied strains. Inhibition of *S. aureus* ATCC 25923 cell growth was observed starting from 15.62 μg/mL. A relevant inhibition of *S. aureus* ATCC 25923 cell growth was observed from 62.5 μg/mL. In the case of *E. coli* ATCC 25922 cells, inhibition of growth was observed from 125 μg/mL. Antibacterial activity was maximal in 20ZnHAp followed by 7ZnHAp compared to control (Figure 16a,b). It was observed that *S. aureus* ATCC 25923 was more sensitive than *E. coli* ATCC 25922 to zinc-doped hydroxyapatite solutions compared to control. 20ZnHAp showed higher activity against *S. aureus* ATCC 25923 compared to 7ZnHAp and control. The growth of *S. aureus* ATCC 25923 and *E. coli* ATCC 25922 cells was not influenced by the presence of pure hydroxyapatite (x_Zn_ = 0) at concentrations ranging from 1000 to 1.95 μg/mL. The results of the antimicrobial assays have emphasized that the best inhibitory activity against the tested microbial strains was achieved for the 20ZnHAp sample, which was also depicted as having the best stable parameter from the tested samples. These studies have revealed that the antimicrobial activity of the tested solutions was influenced both by the solution’s stability and zinc concentration. The antimicrobial activity of ZnHAp solutions is influenced by both the stability of the solutions and the amount of Zn.

According to previous studies [11,12,13,14,61,62], the antimicrobial activity of hydroxyapatite doped with different ions, such as Zn^2+^ and Ag^+^, were strongly influenced by various factors, such as particle size, surface area, surface composition, and its structure. As observed in recent studies [14], the stability of colloidal dispersions is a major factor in cell viability assay. The results of this research have shown that the stability of the dispersions used in this study significantly influences the biocidal effect of ZnHAp. Compared to recent studies [14,61], the results presented in this work revealed a significant decrease in *E. coli* cell growth in the presence of ZnHAp solutions. On the other hand, the antimicrobial activity due to Zn ions present in the HAp structure may be due to the way these ions interact with the microbial membrane, which causes structural changes and permeability [64].

The morphology and stability of these samples were estimated to better understand the stability and aggregation of the ZnHAp with different zinc concentrations (x_Zn_ = 0, x_Zn_ = 0.07, and x_Zn_ = 0.2) in suspensions and to facilitate their targeting to inferior applications in the pharmaceutical, medical, or environmental industries. The studies presented in this paper can help nanomaterials researchers in the process of obtaining and characterizing materials made for applications in different commercial areas according to their unique physical and chemical properties.

## 4. Conclusions

The influence of the stability of the ZnHAp solutions on antimicrobial properties was also evaluated for the first time. The suspension of ZnHAp particles was analyzed by different technics. The nanoparticle size was determined by both SEM studies and DLS measurements, while the stability of the tested solutions was evaluated by ζ-potential and ultrasound-based technique. The results of the Rietveld refining method for the XRD analysis revealed a single phase corresponding to the hexagonal hydroxyapatite. The calculated lattice parameters of HAp, 7ZnHAp, and 20ZnHAp samples from the XRD were also in good agreement with the standard data for the hexagonal hydroxyapatite. FTIR studies highlighted that the spectra of the analyzed samples showed a widening of the peaks and a smoothing with the increase of the zinc concentration. Additionally, particular attention was paid to the colloidal studies of ZnHAp dispersions for the first time by the use of ultrasounds as a technique to characterize dispersion stability. These studies have shown that the stability of ZnHAp solutions is strongly influenced by zinc content. Moreover, the studies presented in this paper have revealed that *S. aureus* ATCC 25923 was more sensitive than *E. coli* ATCC 25922 to zinc-doped hydroxyapatite solutions compared to the control. The development of *S. aureus* ATCC 25923 and *E. coli* ATCC 25922 cells was not influenced by solutions in which x_Zn_ was equal to zero (pure HAp). The biocidal effect of ZnHAp solutions was influenced by both solution stability and Zn content.
